# The Challenge and Importance of Integrating Drug–Nutrient–Genome Interactions in Personalized Cardiovascular Healthcare

**DOI:** 10.3390/jpm12040513

**Published:** 2022-03-22

**Authors:** Ioannis Stouras, Theodore G. Papaioannou, Konstantinos Tsioufis, Aristides G. Eliopoulos, Despina Sanoudou

**Affiliations:** 1 Clinical Genomics and Pharmacogenomics Unit, 4th Department of Internal Medicine, Attikon Hospital Medical School, National and Kapodistrian University of Athens, 11527 Athens, Greece; istouras@gmail.com; 2 Center for New Biotechnologies and Precision Medicine, Medical School, National and Kapodistrian University of Athens, 11527 Athens, Greece; eliopag@med.uoa.gr; 3 First Department of Cardiology, Hippokration Hospital, Medical School, National and Kapodistrian University of Athens, 11527 Athens, Greece; thepap@med.uoa.gr (T.G.P.); ktsioufis@med.uoa.gr (K.T.); 4 Department of Biology, Medical School, National and Kapodistrian University of Athens, 15771 Athens, Greece; 5 Molecular Biology Division, Biomedical Research Foundation of the Academy of Athens, 11527 Athens, Greece

**Keywords:** nutrigenetics, pharmacogenetics, precision medicine, personalized dietary interventions, cytochrome P450

## Abstract

Despite the rich armamentarium of available drugs against different forms of cardiovascular disease (CVD), major challenges persist in their safe and effective use. These include high rates of adverse drug reactions, increased heterogeneity in patient responses, suboptimal drug efficacy, and in some cases limited compliance. Dietary elements (including food, beverages, and supplements) can modulate drug absorption, distribution, metabolism, excretion, and action, with significant implications for drug efficacy and safety. Genetic variation can further modulate the response to diet, to a drug, and to the interaction of the two. These interactions represent a largely unexplored territory that holds considerable promise in the field of personalized medicine in CVD. Herein, we highlight examples of clinically relevant drug–nutrient–genome interactions, map the challenges faced to date, and discuss their future perspectives in personalized cardiovascular healthcare in light of the rapid technological advances.

## 1. Introduction

Despite the tens of thousands of available drugs, major challenges persist in their safe and effective use. Adverse drug reactions (ADRs) are responsible for approximately 5% of hospital admissions, while 10–20% of inpatients suffer from ADRs, which tend to increase both the length of hospitalization and the mortality rate [1,2,3]. ADRs are estimated to be the fourth leading cause of death in hospitalized patients and the cause of approximately 197,000 deaths in the European Union every year, with the total cost of ADRs to society in the EU being approximately EUR 79 billion [4]. However, according to the World Health Organization, as many as 60% of ADRs are preventable [5].

While the vast majority of ADRs refer to drug–drug interactions, drug–nutrient–genome interactions (DNGIs) may pose a considerable risk that is often disregarded in clinical practice [6]. DNGIs can modulate pharmacokinetic (e.g., nutrient affects drug absorption, distribution, metabolism, and excretion) and pharmacodynamic (e.g., nutrient-driven modulation of drug action at a receptor or signaling level) parameters, while the scientific evidence available implicates largely the former [7].

Consequently, dietary elements (including food, beverages, and supplements) are capable of altering drug bioavailability in ways that can drastically affect treatment outcomes. Decreased drug bioavailability may lead to suboptimal drug concentrations at the target tissue and reduced drug effectiveness, while increased bioavailability raises the risk of ADRs and toxicity [8]. Approximately 15% of the patients under medication in the United States also consume herbal products, and among them, 40% experience potential adverse drug–herb interactions [9].

Knowledge of the possible DNGIs is valuable in maximizing the benefits a patient can have from a given treatment while minimizing adverse reactions and therefore represents an integral component of personalized healthcare. The impact of this knowledge is the highest for the most frequently prescribed drugs, which affect the lives of millions of patients worldwide. At the top of this list are drugs used to manage cardiovascular disease (CVD) [10]. Cardiovascular drug ADRs alone account for 10% of all medication-related office visits [11]. Heightened heterogeneity is also reported in patients’ CVD drug responses. The progress in the field of DNGIs, however, has been slow, with a multitude of limitations and challenges lingering. Among them: a shortage of clinical or preclinical evidence on many of the DNGIs for which recommendations are available by different healthcare organizations or drug-related websites, marked heterogeneity in the available recommendations, and erratic use of this information in clinical practice.

This review discusses the primary clinical and preclinical data available on CVD-related DNGIs, their molecular basis, and the role of an individual’s genetic background in this interplay. Emphasis is given on current limitations, challenges, and opportunities in this field through the prism of personalized medicine.

## 2. Drug–Nutrient Interactions Related to CYP Genes

The cytochrome P450 (CYP) superfamily consists of enzymes crucial for phase I first-pass drug metabolism reactions (or pre-systemic clearance), which take place mainly in the intestine and the liver. These include processes of oxidation, reduction, and hydrolysis. Furthermore, CYP enzymes participate in the biotransformation of prodrugs to their active forms [12]. Overall, CYP enzymes are involved in approximately 75% of drug metabolic reactions [13,14]. In the human genome, 18 CYP families and 57 CYP genes have been identified with CYP1, CYP2, CYP3, and CYP4 being the most relevant enzyme families concerning drug metabolism [15]. Among them, CYP3A4 is the most abundantly expressed and has been implicated in the metabolism of over 50% of the currently available clinical pharmaceuticals and xenobiotics, including a large number of cardiovascular drugs [16].

The expression and/or activity of CYP enzymes can be significantly modulated by different drugs, nutrients, and genetic polymorphisms. Drugs can serve as substrates, inducers, or inhibitors of CYP enzymes, with one CYP-one drug, one CYP-multiple drugs, or multiple CYP-one drug associations. A similar pattern gradually emerges concerning the association of CYP enzymatic activity and different nutrients. For example, CYP1A2 is responsible for more than 95% of caffeine metabolism; carriers of the CYP1A2 rs762551 AA genotype are fast metabolizers, tend to consume more coffee, and display reduced appetite and energy compared to slow caffeine metabolizers who carry the CC genotype [17]. Such associations can have a significant impact on drug pharmacokinetics, ultimately resulting in clinically relevant DNGIs. A considerable number of CVD DNGIs have been associated with CYP enzymes, especially CYP3A4 and CYP1A2.

### 2.1. Antihypertensives—Calcium Channel Blockers

Calcium channel blockers (CCBs) are well-established and generally well-tolerated antihypertensive agents. CCBs can be distinguished in two classes, namely dihydropyridines and non-dihydropyridines. Dihydropyridine CCBs act by blocking predominantly L-type calcium channels. Despite having the same basic mechanism of action and similar chemical structure, different dihydropyridines present with different pharmacokinetic profiles (e.g., the elimination half-time, the extended-release mechanism of some formulations, and the lipophilicity of the different molecules) and diverse effects on BP, heart rate (HR) and left ventricular mass. Diet appears to affect individual responses, resulting in marked inter-individual variability. Among the different dietary components, grapefruit juice has attracted the most attention and has been associated with increased plasma concentrations (C_max_) and area under the curve (AUC) when consumed together with most drugs in this category [18]. C_max_ and AUC are widely used pharmacokinetics measures of the maximum serum concentration that a drug achieves and the drug amount reaching an individual’s bloodstream in a given time period, respectively, after dosing.

Representative examples and the lessons learned from different studies are presented below.

#### 2.1.1. Nifedipine

Nifedipine is a well-known dihydropyridine, used to treat hypertension and angina pectoris. It undergoes extensive first-pass metabolism [19], predominantly by the CYP3A4 isoenzyme in the liver and intestinal mucosa [20]. Since CYP3A4 can be modulated by numerous nutrients, nifedipine’s association with CYP3A4 has driven multiple studies into possible DNGIs. A prime example is a clinical study of the consumption of St. John’s wort (SJW), a potent inducer of CYP3A4, on nifedipine treatment. Specifically, SJW administration in healthy subjects significantly decreased the area under the concentration-time curve (AUC_0–∞_) (reflecting the actual body exposure to the drug after administration of a dose of the drug) for nifedipine, concomitantly with a significant increase in the AUC_0–∞_ for its metabolite, dehydronifedipine. This effect was shown to be mediated through the induction of CYP3A4, which was modulated by the PXR transcription factor [21]. Importantly, in individuals carrying specific PXR genetic variants, the effect of SJW on nifedipine was significantly different. Specifically, individuals with the H1/H1 genotype had weaker basal transcription of the CYP3A4 gene, but much higher SJW-inducible metabolic activity of CYP3A4 than subjects with H1/H2 and H2/H2 [22]. These findings demonstrate both the significant impact of nutrients on cardiovascular drug response and the relevance of the genetic background in modulating further this interaction in what could be considered a triangle of tightly interconnected parameters ultimately governing patient drug response.

*Ginkgo biloba* is a supplement investigated for its putative effects on nifedipine response. At the clinical level, *Ginkgo biloba* consumption has been reported to increase nifedipine maximal plasma concentration in some individuals by as much as two-fold, with subsequent presentation of ADRs, including headache, dizziness, hot flushes, and tachycardia [23]. Nevertheless, the nifedipine pharmacokinetic changes did not reach statistical significance in the study group (*n* = 10 individuals) [23], likely reflecting genetic influences. In an in vivo animal study, rats treated with *Ginkgo biloba* extract and oral nifedipine showed a statistically significant 1.6-fold increase in nifedipine maximal plasma concentration and AUC and a 2.1-fold increase in absolute drug bioavailability [24]. The respective pharmacokinetic parameters for intravenously administered nifedipine were not altered, which supports the hypothesis that *Ginkgo biloba* slows down the first-pass metabolism of orally administered nifedipine, probably by inhibiting CYP3A4-mediated oxidation [24].

Nifedipine pharmacokinetics has further been reported to be affected by co-ingestion of grapefruit juice. Grapefruit is a well-established inhibitor of CYP3A4. It leads to irreversible inactivation of intestinal CYP3A4, resulting in reduced presystemic metabolism and increased oral bioavailability of drugs that are largely metabolized through this pathway [25]. The recovery of intestinal CYP3A4 activity after a normal single exposure to grapefruit juice has a half-time of about 24 h and is complete within 3 days, while large doses of grapefruit juice may also inhibit hepatic CYP3A4 [26,27]. Although most dihydropyridine CCBs are metabolized by CYP3A4, the direct evidence on grapefruit’s role in patients’ response is limited to only a few studies for specific drugs. In regard to nifedipine specifically, multiple clinical studies have shown that grapefruit juice significantly decreases its metabolism by increasing its AUC and bioavailability [28,29]. This effect was observed following oral, but not intravenous, administration of nifedipine, and it appears to be dampened when lower amounts of grapefruit juice were administered (250 mL vs. 400 mL or 500 mL) [30]. Notably, both inter-individual variability [30], as well as a significant inter-population variability (e.g., Caucasians and South Asians) [28], have been reported to modify the grapefruit juice effect on nifedipine metabolism. This variability could be at least partly explained by genetic variation in the CYP3A4 gene and consequently the CYP3A4 protein levels and/or enzymatic activity [31]. In line with this notion, a meta-analysis of the worldwide distribution of major CYP alleles demonstrated that CYP3A4 had the highest inter-population variability, and among different populations, the greatest difference was observed between the CYP3A4 alleles carried by Caucasians and South Asians [32].

Results from in vivo and in vitro investigations in rats showed that among the most important grapefruit furanocoumarin derivatives—bergaptol, bergamottin, and 6′,7′-dihydroxybergamottin—bergamottin had a major inhibitory action on CYP3A4 and led to a significant increase in the AUC following intraduodenal administration of nifedipine [33]. Furthermore, UV-irradiated or heat-treated grapefruit juice, both of which drastically reduce the concentrations of bergamottin, 6′,7′-dihydroxybergamottin, and bergaptol, did not influence nifedipine pharmacokinetics following intraduodenal administration in rodents [34,35]. The available information on nifedipine–grapefruit interactions has led healthcare organizations, such as Mayo Clinic and the United Kingdom National Health Service (NHS), to advise against their concomitant consumption [36,37]. In summary, nifedipine metabolism can be significantly affected by different nutrients, with the major ones investigated thus far being SJW, Ginkgo biloba, and grapefruit juice. These interactions are likely to be mediated, at least in part, by the CYP3A4 enzyme, and considering the multiplicity of its alleles, subject to considerable genetically driven heterogeneity.

#### 2.1.2. Felodipine

Felodipine has a well-proven effect in the treatment of hypertension and angina pectoris. The predominant step of felodipine metabolism is the CYP3A4-mediated oxidation of the dihydropyridine ring, resulting in a pyridine derivative (dehydrofelodipine) that is devoid of calcium channel blocking activity [38,39]. Findings from multiple clinical studies on healthy individuals consistently and reproducibly demonstrated a significant increase in AUC, C_max,_ and bioavailability of orally administered felodipine when consumed together with grapefruit juice, whereas there was no alteration when felodipine was administered intravenously. These pharmacokinetic alterations were accompanied by clinically augmented hemodynamic effects, mainly on diastolic BP reduction and HR increase, as well as vasodilation-related side effects, such as headaches, facial flushing, and lightheadedness [40,41,42]. Similar observations were reported for borderline hypertensive men [29]. These effects were enhanced in elderly individuals, with the concomitant consumption of grapefruit juice (250 mL) and felodipine (2.5 mg), leading to increased AUC and C_max_ by 2.9-fold and 4-fold, respectively [43]. These findings could be explained by the strong inhibition of CYP3A4 by the grapefruit furanocoumarin derivative bergamottin [33]. Accordingly, the use of furanocoumarin-free grapefruit juice did not affect felodipine AUC and C_max_, pointing to furanocoumarins as the main active ingredients of grapefruit responsible for the inhibition of felodipine, along the lines of nifedipine findings [44]. Notably, the effect of grapefruit juice was significant even when it preceded the oral administration of felodipine by up to 24 h, although the interaction was greater with decreasing time intervals [42]. These observations underscore the need to refrain from grapefruit juice consumption at least 24 h before and during felodipine treatment.

#### 2.1.3. Amlodipine

Amlodipine is one of the most commonly prescribed dihydropyridine CCB for hypertension and ischemic heart disease. Once in the human body, it undergoes dehydrogenation to a pyridine derivative, with CYP3A4 shown to play a key role in this process [45]. The DNGIs of amlodipine have been investigated only to a limited extent. At the clinical level, co-administration of grapefruit juice and amlodipine did not show a consistently significant effect across studies [18,46,47]. Nevertheless, this DNGI should not be neglected, as variations among patient response (including CYP3A4 polymorphisms) and a higher intake of grapefruit juice could result in a pronounced effect [48]. The recommendations by healthcare organizations on the concomitant consumption of amlodipine and grapefruit are variable. The UK NHS recommends to patients receiving amlodipine: “Do not eat or drink lots of grapefruit or grapefruit juice while you’re taking this medicine. Grapefruit can increase the concentration of amlodipine in your body and worsen side effects” [49]. Drugs.com states: “The consumption of grapefruit juice may slightly increase plasma concentrations of amlodipine” and “monitoring for calcium channel blocker adverse effects (e.g., headache, hypotension, syncope, tachycardia, edema) is recommended” [50]. However, other organizations (e.g., Mayo Clinic) do not alert patients to the potential of such an interaction [51].

#### 2.1.4. Manidipine, Nisoldipine, Nicardipine, Nitrendipine

Manidipine is a long-lasting dihydropyridine CCB, with several traits that make it unique in its class [52]. Although there is a gap in the literature on the exact catabolic pathways of manidipine, it is assumed that CYP3A4 is involved, similarly to other dihydropyridine CCBs. A clinical study confirmed the grapefruit juice-mediated CYP3A4 inhibition, and notably demonstrated that this was distinct for the two manidipine enantiomers, resulting in altered stereoselective disposition of the drug [53]. Of note, since there was no effect on the elimination half time, indicating there was little inhibition of hepatic CYP3A4 activity, this inhibitory effect is expected to take place in the small intestine [53].

Similarly, a limited number of clinical trials has shed light on DNGIs of other dihydropyridine CCBs, such as nisoldipine, nicardipine, and nitrendipine. Concomitant nisoldipine (5 mg per os) and grapefruit juice (200 mL) consumption resulted in significant increases in nisoldipine plasma concentration, which were accompanied by enhanced hemodynamic alterations, as reflected by significant systolic and diastolic BP decreases and side effects, such as headaches in 75% of patients. These effects persisted for up to 3 days after nisoldipine and grapefruit juice co-administration [54]. Grapefruit juice has been reported to also affect response to nicardipine, in the form of transient reflex tachycardia development. This could at least in part be associated with the effect of grapefruit on the stereoselective metabolism of nicardipine, slightly in favor of (+)-nicardipine compared to (--nicardipine [55]. Scarce evidence on nitrendipine DNGIs in humans suggests an effect of grapefruit on AUC increase, while in vivo animal studies showed an effect of pomegranate juice, a potent CYP3A inhibitor, on intestinal CYP3A4 activity, AUC, and maximal plasma concentration [56,57,58]. Overall, although no official recommendations about these dihydropyridine CCBs and grapefruit or pomegranate interactions are currently available, healthcare professionals should be aware of possible DNGIs and should advise patients to exert caution when consuming grapefruit or pomegranate while on these medications.

### 2.2. Lipid-Lowering Drugs, HMG-CoA Reductase Inhibitors

Statins are competitive inhibitors of 3-hydroxyl-3-methylglutaryl coenzyme A (HMG-CoA) reductase, a rate-limiting enzyme of cholesterol biosynthetic pathway, which catalyzes the conversion of HMG-CoA to mevalonate [59]. They also promote the upregulation of the low-density lipoprotein (LDL) receptor, as well as the decrease in apolipoprotein B (ApoB)-containing lipoproteins [60,61]. These events are responsible for a significant decrease in LDL cholesterol (LDL-C), which is involved in the pathophysiology of atherosclerosis [62,63]. Statins are administered either as lactone prodrugs or as the active acidic form [64].

#### 2.2.1. Simvastatin

Simvastatin is the most widely used cholesterol-lowering medication according to the US Center for Disease Control (CDC). Its metabolism is dependent on CYP3A4, which modulates the oxidation of simvastatin acid and statin lactone to open-chain acid and lactone metabolites [60]. Consequently, nutrients enhancing or inhibiting CYP3A4 expression and/or enzymatic activity can influence response to treatment. As expected, grapefruit has been shown to have a significant inhibitory effect on simvastatin metabolism by increasing simvastatin and simvastatin acid C_max_ and AUC_0–24 h_ by >3.3- to 4.3-fold [65]. The mechanism proposed to regulate this interaction entails the inhibition of CYP3A4-mediated first-pass metabolism of simvastatin in the gut by furanocoumarins. Thus, enhanced simvastatin bioavailability may promote its cholesterol-lowering effect, but also exacerbate its side effects. In a rare case, rhabdomyolysis was described in a 40-year-old woman on simvastatin who consumed one grapefruit per day [66]. Additional clinical trials are needed to assess the effects of grapefruit and other dietary components with CYP3A4 inhibitory action, concerning rare but serious simvastatin ADRs such as pulmonary toxicity [67]. Simvastatin clearance is also reduced by the green tea component epigallocatechin-3-gallate (EGCG) [68], which inhibits both CYP3A4 and hepatic organic anion transporting polypeptides (OATPs) 1B1 and 1B3 (discussed in Section 3), leading to increased circulating drug levels in rats [69].

Although experimental data suggest that pomegranate exerts an inhibitory effect on CYP3A4 similarly to grapefruit, a study in healthy volunteers did not observe a similar effect when pomegranate was consumed together with simvastatin. This observation highlights the need for in-depth clinical assessment of each DNGI rather than assuming identical interactions for all drugs or all nutrients of a given category [70].

St. John’s wort has been shown to directly interfere with simvastatin metabolism in multiple clinical studies of healthy and hypercholesterolemic individuals. As anticipated, it led to a significant decrease in simvastatin hydroxy acid AUC_0–24 h_, as well as simvastatin and simvastatin hydroxy acid concentrations, resulting in a significantly reduced therapeutic effect [71,72].

Capsaicin, the main spicy component of different species of hot peppers (e.g., red chili, jalapeños, habaneros) [73], has also been proposed to decrease simvastatin efficacy in rats, via CYP3A4 induction [74]. Further studies are needed to validate these observations in humans.

#### 2.2.2. Atorvastatin

Atorvastatin is the second most frequently used cholesterol-lowering medication according to the CDC. Its metabolism to the active metabolites 2-hydroxy atorvastatin and 4-hydroxy atorvastatin implicates CYP3A4-mediated oxidation [75]. Similar to simvastatin, grapefruit and SJW have been shown to significantly alter atorvastatin pharmacokinetics in multiple studies. Specifically, grapefruit significantly increased atorvastatin acid and atorvastatin lactone AUC_0–72 h_ by as much as 330%, while decreasing 2-hydroxy atorvastatin acid and 2-hydroxy atorvastatin lactone AUC_0–72 h_ [76,77,78], without, however, clinically evident alterations in liver function or creatine phosphokinase (CPK) levels [79]. SJW significantly increased LDL-C serum levels and total cholesterol in hypercholesterolemic patients, without, however, significantly affecting high-density lipoprotein cholesterol (HDL-C) or triglycerides [80].

#### 2.2.3. Rosuvastatin

Rosuvastatin is another drug of the class of statins known to be metabolized via CYP2C9 and CYP2C19 and to a lesser extent CYP3A4 [81]. Original research data on rosuvastatin DNGIs is limited. Although a case report suggested that the combination of rosuvastatin and pomegranate juice could have been the cause for the development of rhabdomyolysis [82], current recommendations by the NHS are “unlike with other statins, such as simvastatin and atorvastatin, it’s safe to drink grapefruit juice with rosuvastatin” [83].

### 2.3. Antiarrhythmics

Antiarrhythmic agents are categorized in four distinct classes based on their mode of action: class I—sodium channel blockers; class II—beta blockers; class III—potassium channel blockers; and class IV—calcium channel blockers. In general, they have a narrow therapeutic index, and thus, they are often susceptible to interactions that can cause significant ADRs. In this regard, dietary components merit special consideration in the clinical application of antiarrhythmics. Polymorphisms in the genes encoding drug-metabolizing enzymes should also be considered, as they have been implicated in inter-individual variability in antiarrhythmic drug efficacy and toxicity. This section focuses on representative examples from different classes of antiarrhythmic drugs.

The class IV antiarrhythmic drugs diltiazem and verapamil, as well as amiodarone (class III) and quinidine (class IA), are all metabolized by CYP enzymes and have been shown to interact with grapefruit juice. These interactions have been assessed in human studies and have been shown to result in significantly increased AUC and/or C_max_, and in some cases, significant electrophysiological alterations, including PR interval prolongation [84,85,86,87]. The magnitude of the DNGI effect has been proposed to vary considerably depending on drug bioavailability. For example, grapefruit has a significant, yet limited, effect on quinidine metabolism, which is likely a consequence of the high bioavailability of orally administered quinidine (70%) compared to other drugs (e.g., 20–30% for verapamil, 40% for diltiazem) that renders first-pass metabolism less significant in total quinidine bioavailability [88,89]. Inter-individual variations in the magnitude of antiarrhythmic DNGI effects have been reported and are likely influenced by genetic variability in CYP family genes that impact enzymatic activity and/or expression levels [84,90]. This may explain a case report of an 83-year old woman developing atrial fibrillation following grapefruit consumption while on amiodarone medication [91].

Assessment of pharmacokinetic interactions between caffeine and the anti-arrhythmic drugs verapamil, propafenone, and mexiletine is also required, as they are all metabolized by CYP1A2, an enzyme with pronounced inter-individual genetically-driven variation in activity. Sporadic evidence supports the impact of these interactions. For example, the increased plasma concentrations of caffeine observed following administration of mexiletine in some patients may reflect DNGIs responsible for ADRs in patients with cardiac arrhythmia [92].

### 2.4. Anticoagulants

Warfarin belongs to the drug class of anticoagulants and is metabolized by a variety of P450 isoenzymes, including CYP1A1, CYP1A2, CYP2C9, CYP2C19, and CYP3A4 [93]. This major CYP involvement renders warfarin metabolism susceptible to multiple DNGIs. A variety of clinical studies and case reports have implicated different nutrients in warfarin response. Representative examples include SJW and caffeine, each of which induces multiple CYP enzymes, leading to a significantly decreased international normalized ratio (INR) and subtherapeutic coagulation [94]. The INR is a system established for reporting the results of blood coagulation tests. Conversely, case reports have suggested that cranberry products, grapefruit juice, pomegranate juice, mangos, goji berries, milk thistle, and cannabis each inhibit one or more of the CYP3A4, CYP1A2, and CYP2C9 enzymes, and have been shown to result in elevated INR in patients receiving warfarin, supratherapeutic anticoagulation, and in some cases, severe bleeding requiring hospitalization [95,96,97]. However, clinical trials have only been published for cranberry and grapefruit juice, and no significant interactions with warfarin were observed [97].

Beyond CYP-mediated DNGIs, warfarin has notable pharmacodynamics-related DNGIs, involving vitamin K epoxide reductase complex subunit 1 (VKORC1). Vitamin K–rich products such as green, leafy vegetables, and especially vitamin K-containing supplements, can decrease warfarin activity leading to reduced INR, which in turn increases the patient’s risk for suboptimal anticoagulation, and potentially thromboembolic events such as deep venous thrombosis, pulmonary embolism, myocardial infarction, or stroke [98]. Polymorphisms in VKORC1 have been shown to modulate the interaction of warfarin and vitamin K, with the presence of specific alleles necessitating modification of both warfarin dose and vitamin K intake [99]. However, older recommendations for diets low in vitamin K as appropriate for warfarin-treated patients should now be considered outdated. Instead, once empirical or genotype-based warfarin dose titration is accomplished in each patient, their usual dietary pattern needs to be carefully maintained, and any planned changes in diet or multivitamin usage should be reported to their doctor [100].

## 3. P-Glycoprotein-Related DNGIs

P-glycoprotein (PGP) plays a key role in drug absorption and disposition in the intestinal and, to a lesser extent, the liver and the kidneys. PGP controls the cellular excretion of a variety of drug molecules, many of which are later reabsorbed. As a result, by regulating the mean residence time of a drug molecule inside the cell, PGP also controls drug exposure to drug-metabolizing enzymes [96].

PGP is encoded by the ABCB1 gene, whose expression is regulated by the highly polymorphic transcription factor Pregnane X Receptor (PXR). Alterations in its activity due to genetic variations, certain drugs, and/or nutrients can significantly compromise the therapeutic outcome of a PGP metabolized drug. Notably, at least 28 single nucleotide polymorphisms (SNPs) have been identified that can modify PGP function [101] and may partly explain the reported inter-individual variability in drug pharmacokinetics and toxicity [96]. At the dietary level, multiple nutritional components across food groups, such as fruit juices, spices, herbs, cruciferous vegetables, and green tea, have been shown to modulate drug bioavailability through PGP regulation [102]. Most of these data, however, have been derived from in vitro studies. In the CVD setting, studies on PGP-related DNGIs are lagging behind those relating to CYP enzymes. Representative examples are presented below.

### 3.1. Digoxin

Digoxin is a drug of choice in congestive heart failure. It is a cardiac glycoside acting on Na^+^/K^+^ ATPase, with positive inotropic action on the heart muscle. It is transported by PGP and has a narrow therapeutic window. Two clinical studies demonstrated that SJW consumption significantly decreases digoxin AUC and C_max_ in a dose-dependent manner, therefore reducing drug efficacy [103,104]. This effect is likely to be associated with the hyperforin concentration in SJW, as hyperforin regulates PGP expression via its interaction with PXR [105]. Caution should be exercised during SJW discontinuation, since increased toxicity may precipitate during this process [103].

### 3.2. Talinolol

Talinolol is a selective b1-adrenergic receptor blocker and substrate of PGP [106]. Different in vitro and in vivo animal studies strongly support the interaction of talinolol with grapefruit juice and its ingredients [107,108]. Experimental data in mice showed a significantly increased AUC and C_max_ for R- and S-talinolol following co-administration of grapefruit juice, possibly due to impaired intestinal secretion of talinolol. At the clinical level, co-administration of talinolol and grapefruit juice significantly decreased talinolol AUC and C_max_, without affecting ABCB1 mRNA or PGP levels at the duodenum [109]. These exactly opposite observations in the effect of grapefruit on PGP-regulated digoxin bioavailability between humans and rats have been attributed to the different affinities of naringin, a flavonoid of grapefruit juice, for human and rat PGP [110]. Importantly, however, it serves as a reminder of the significant inter-species differences that hinder the untroubled extrapolation of animal DNGI findings to humans.

### 3.3. Quinidine and Diltiazem

PGP is also involved in the pharmacokinetics of the anti-arrhythmic drugs quinidine and diltiazem, in addition to CYP enzymes, with limited yet significant DNGI data from in vitro and in vivo animal studies. Specifically, green tea has been shown to significantly enhance quinidine absorption in the ileum of rats, possibly due to a catechin-driven suppression of quinidine efflux via PGP [111]. Piperin pretreatment significantly reduced diltiazem bioavailability in rats, possibly through PXR activation and intestinal PGP induction [112]. However, the clinical significance and the safe extrapolation of these results in humans require further investigation.

## 4. Organic Anion-Transporting Polypeptides (OATPs)-Related DNGIs

Organic anion-transporting polypeptides (OATPs) are transporters involved in the uptake of multiple clinically important drugs from the bloodstream into cells, thereby modulating pharmacokinetic properties [113]. Several dietary components have been found to confer a significant effect on the function of different OATPs. For example, flavonoids, which are present in a broad range of different fruits and vegetables (including citrus fruits, berries, grapes, apples, corn), as well as quercetin (present in fruits, vegetables, leaves, and grains) and licorice root (frequently used flavoring and sweetening agents in foods, beverages, candies, and dietary supplements) exert a significant inhibitory effect on OATP1B1 and/or OATP2B1 [114,115,116]. Importantly, multiple genetic variants, including SNPs and copy number variants, have been identified in the OATP genes and shown to modulate protein function [117]. Consequently, OATPs are anticipated to play a significant role in drug–nutrient interactions as well as DNGIs. However, the number of clinical and animal studies investigating these relationships is still limited to enable reaching firm conclusions.

### Aliskiren

The anti-hypertensive drug aliskiren is substrate of OATP2B1 and OATP1A2 [118,119]. Clinical studies involving administration of grapefruit juice concomitantly with aliskiren demonstrated significantly decreased drug AUC_0–∞_ and C_max_ by as much as 61% and 81%, respectively, possibly due to the inhibitory effect of the grapefruit component naringin on the hepatic OATP1A2 and/or the OATP2B1-mediated inhibition of aliskiren uptake by the small intestine [119,120]. Furthermore, a significant effect was observed when apple or orange juice was co-administered with aliskiren to healthy volunteers: C_max_ was reduced by 80% and 84%, and the AUC by 62% and 63%, respectively. The reduction of aliskiren oral bioavailability was accompanied by a higher plasma renin activity by 87% and 67% for orange and apple juice consumption, respectively. In vitro studies demonstrated the inhibition of OATP1A2 by hesperidin and OATP2B1 by tangeritin and nobiletin, all compounds of orange juice. Correspondingly, quercetin and kaempferol, found in apple juice, have shown in vitro inhibition of OATP2B1 [121]. Taken together, grapefruit, orange, and apple juice exert an inhibitory action on OATP1A2 and OATP2B1, and co-administration with aliskiren should be best avoided. This is reflected in a relevant recommendation by drugs.com (accessed on 15 February 2022), stating that “you should avoid drinking orange, apple, or grapefruit juice as much as possible during treatment with aliskiren; studies have shown that drinking these juices regularly or within a short period before or after a dose of aliskiren can interfere with the absorption of the medication” [122]. Similar recommendations have been produced by the National Institute for Health and Care Excellence (NICE) [123].

## 5. Current Challenges

The evidence reviewed herein highlights the major roles of DNGIs in CVD drug responses. Although the impact of DNGIs is gradually increasing, the pace of generation of new knowledge remains slow, and its integration in routine clinical practice and daily life is even slower. For example, only a limited number of the known CYP3A4-modulating nutrients have been assessed to date in relation to CVD drugs [124]. Considerable additional work is therefore needed toward a comprehensive map of nutrients interfering with cardiovascular drug response via modulation of CYP enzymes. Furthermore, the large number of different alleles (>20) identified for CYP3A4, as well as other cardiovascular drug-relevant genes, in combination with their highly variable frequency in different populations, necessitate the validation of findings in large, well-characterized, and multi-ethnic cohorts [32].

The currently available information is likely to be only the tip of the iceberg, with numerous challenges awaiting to be overcome to achieve a much-needed breakthrough. First, the expected large number of possible interactions among different nutrients, drugs, and genetic variations results in overwhelming complexity. Therefore, they cannot all realistically be assessed in clinical or in vivo animal studies. Furthermore, as the example of talinolol showed, DNGIs can be different or even opposite between humans and other species. In vitro approaches could be employed for high-throughput screening of DNGIs; however, the findings would still serve as an indication rather than confirmation. In vitro findings and results from in silico prediction tools commonly feed into educated assumptions of DNGIs among different members of the same drug class and groups of dietary components which, however, may not translate to clinically relevant effects.

A second set of challenges relates to the marked heterogeneity in study designs, which often renders the comparison of findings across studies difficult or even impossible to implement and hinders progress altogether. The establishment of a widely accepted framework for the design and implementation of DNGI screening studies could help toward ensuring high/consistent research standards and comparability of data. 

Another major obstacle is the access to comprehensive, well-organized, evidence-based information on DNGIs. In the majority of cases, the information is fragmented, with a focus on specific drugs, nutrients, proteins (e.g., CYP3A4, OATP2B1), or selected DNGIs, and the scientific evidence to support the described DNGIs is elusive. The aforementioned fragmentation of information and the utilization of inferred predictions inevitably lead to contradictory information provided by different sources or databases.

A fourth set of challenges relates to the lack of an official classification system for DNGIs, rendering the translation of the scientific knowledge into clinical practice problematic. For example, some DNGIs may be relevant only for high amounts of specific nutrients, and/or specific genetic variants. Other DNGIs may be associated with severe phenotypes and require an emphasis to be given when communicating dietary recommendations to patients initiating specific drug treatments. Toward this direction, drugs.com (accessed on 15 February 2022) classifies drug interactions in four categories: major, moderate, minor, or unknown, thus facilitating clinical implementation.

Finally, there are no official guidelines on the dietary recommendations that patients on specific medication should receive. The options currently provided are “use” or “avoid”. However, this could potentially lead to a highly restricted dietary plan and subsequently to a compromised quality of life, especially when the DNGIs involve nutritional components present in a broad range of foods, in frequently consumed foods, or when multiple drugs involving multiple DNGIs are co-administered.

## 6. Future Perspectives

Mapping the challenges is the first step toward overcoming them. In the era of increasingly personalized medicine approaches, the triangle of nutrient–drug–genome interactions should and can be studied in far greater breadth and depth. Systematic and ideally large-scale and high-throughput approaches are required. Organs-on-a-chip and 3D organoids are rapidly evolving and could play a central role in the process [125,126,127]. A more uniform, carefully selected, and widely accepted framework relating to study designs should be adopted to test DNGIs in large cohorts and different populations. Toward this goal, the rapid advances in omics technologies, the opportunities provided by access to “big data” (such as the UK Biobank), the tremendous capabilities and applications of the Internet of Things, along with cutting-edge machine learning approaches and artificial intelligence (AI) tools offer unprecedented opportunities.

Looking into the not-so-distant future, the pioneering concept of a “Virtual Digital Twin” [128] could explore the patient’s genetic profile, dietary habits/preferences, frequently used dietary supplements, and prescribed drugs along with the latest dietary recommendations for different DNGIs, to offer highly personalized and all-encompassing health guidance [128].

However, the successful and timely incorporation of a new model of patient care into routine clinical practice will not be a trivial task. Appropriate clinical practice guidelines will need to be established and widely communicated to all healthcare providers (e.g., medical doctors, nursing staff, and clinical nutritionists). Comprehensive, up-to-date DNGI databases should be freely and easily accessible. Public awareness campaigns will be important to enhance doctor–patient communication and patient compliance. Of note, approximately 38 million adults in the US use herbal products or other natural supplements, but only one-third inform their physician, primarily due to the misconception that herbal products are “natural and therefore safe” [129,130].

## 7. Conclusions

DNGIs compromise the safety and efficacy of CVD drugs. Although there are multiple examples of clinically proven interactions, this field remains largely unexplored. The application of truly personalized medicine in CVD, however, will require a profound understanding of DNGIs. Drug administration along with genetically guided nutritional advice would directly impact CVD patient quality of life and ease the burden of unsuccessful treatments on doctors and healthcare systems. The benefits would be anticipated to be further magnified for drugs with a narrow therapeutic index and dose titration requirements, where even small changes in dose–response effects can have great consequences. Awareness of DNGIs may also help to improve patients’ compliance since ADRs are a leading cause of non-compliance. The groundbreaking scientific and technological advancements of recent years offer unprecedented opportunities toward this direction, rendering the integration of finely mapped dietary and genetic parameters in the therapeutic algorithms of CVD a tangible goal.

## Data Availability

Not applicable.
